# Heparin Enriched-WPI Coating on Ti6Al4V Increases Hydrophilicity and Improves Proliferation and Differentiation of Human Bone Marrow Stromal Cells

**DOI:** 10.3390/ijms23010139

**Published:** 2021-12-23

**Authors:** Davide Facchetti, Ute Hempel, Laurine Martocq, Alan M. Smith, Andrey Koptyug, Roman A. Surmenev, Maria A. Surmeneva, Timothy E. L. Douglas

**Affiliations:** 1 Engineering Department, Lancaster University, Lancaster LA1 4YW, UK; l.martocq@lancaster.ac.uk; 2 Department of Chemistry, Molecular Sciences Research Hub, Imperial College London, London W12 0BZ, UK; 3 Institute of Physiological Chemistry, Technische Universität Dresden, 01307 Dresden, Germany; hempel-u@msx.tu-dresden.de; 4 Department of Pharmacy, School of Applied Sciences, University of Huddersfield, Huddersfield HD1 3DR, UK; a.m.smith@hud.ac.uk; 5 Department of Quality Technology, Mechanical Engineering & Mathematics, Mid Sweden University, 831 25 Ostersund, Sweden; andrey.koptyug@miun.se; 6 Physical Materials Science and Composite Materials Centre, Research School of Chemistry & Applied Biomedical Sciences, National Research Tomsk Polytechnic University, 634050 Tomsk, Russia; rsurmenev@mail.ru (R.A.S.); surmenevamaria@mail.ru (M.A.S.); 7 Piezo- and Magnetoelectric Materials Research & Development Centre, Research School of Chemistry & Applied Biomedical Sciences, National Research Tomsk Polytechnic University, 634050 Tomsk, Russia; 8 Materials Science Institute (MSI), Lancaster University, Lancaster, UK

**Keywords:** WPI fibrils, Ti6Al4V, additive manufacturing, osseointegration, heparin, tinzaparin, osteoblast differentiation, coating, enriched

## Abstract

Titanium alloy (Ti6Al4V) is one of the most prominent biomaterials for bone contact because of its ability to bear mechanical loading and resist corrosion. The success of Ti6Al4V implants depends on bone formation on the implant surface. Hence, implant coatings which promote adhesion, proliferation and differentiation of bone-forming cells are desirable. One coating strategy is by adsorption of biomacromolecules. In this study, Ti6Al4V substrates produced by additive manufacturing (AM) were coated with whey protein isolate (WPI) fibrils, obtained at pH 2, and heparin or tinzaparin (a low molecular weight heparin LMWH) in order to improve the proliferation and differentiation of bone-forming cells. WPI fibrils proved to be an excellent support for the growth of human bone marrow stromal cells (hBMSC). Indeed, WPI fibrils were resistant to sterilization and were stable during storage. This WPI-heparin-enriched coating, especially the LMWH, enhanced the differentiation of hBMSC by increasing tissue non-specific alkaline phosphatase (TNAP) activity. Finally, the coating increased the hydrophilicity of the material. The results confirmed that WPI fibrils are an excellent biomaterial which can be used for biomedical coatings, as they are easily modifiable and resistant to heat treatments. Indeed, the already known positive effect on osteogenic integration of WPI-only coated substrates has been further enhanced by a simple adsorption procedure.

## 1. Introduction

Ti6Al4V is a well-known biomaterial for orthopaedic implants widely used due to its high specific strength and high corrosion resistance. One of the advantages of this material is that it can be produced using AM techniques, allowing the generation of implants with complicated shapes while keeping production costs reasonably low [1]. The stability and long-term success of an implant depends on its ability to osseointegrate after its stable fixation to the surrounding bone. One of the known methods of facilitating this process is by modifying the surface of the implant with a coating. Collagen is the most studied protein for this type of application, especially in its fibrillar form [2,3,4]. The advantages of using protein fibrils lie in their high surface/volume ratio, which facilitates their adsorption onto the substrate, and the possibility of incorporating other molecules on their surface such as phenols [4,5] and marine polysaccharides [5]. Heparin is a highly sulfated glycosaminoglycan (GAGs) widely used as a coating for implants due to its ability to accumulate and release growth factors (in the form of a crosslinked hydrogel), improve blood compatibility by reducing the inflammatory and coagulative response, and facilitate bone cells’ adhesion, growth and osteogenic differentiation [6].

Previous studies demonstrated that WPI increases cell proliferation and osteogenic differentiation in soluble form both in cell culture medium [7] and as a hydrogel [8]. Additionally, this compound shows antibacterial effects [9]. WPI consists of more than 75% β-lactoglobulin (β-lg) and under conditions of acid hydrolysis (pH < 3, T > 80 °C) it forms uniform fibrils that are several micrometers long and a few nanometers thick. The fibrillation process occurs thanks to peptides that self-assemble into amyloid-like structures [10]. In the fibrillar form, they have supported the attachment, spreading and differentiating of human bone marrow stromal cells (hBMSC), which are cells with great clinical relevance for bone regeneration. WPI fibrils also proved to be resistant to autoclaving, making them an excellent option for biomedical coatings which need to be sterile [11].

In this study, we demonstrated the possibility of coating Ti6Al4V with heparin-enriched WPI fibrils in order to combine the positive effects of all the components, resulting in a system that is easily sterilizable and can lead to the production, via AM, of complex-shaped prostheses that are highly biocompatible. In addition, we evaluated the influence of the molecular weight of heparin on its ability to modulate the growth of hBMSC by comparing the effects of sodium heparin (MW: ~20,000 Da) and tinzaparin (LMWH; MW: ~8000 Da). For this purpose, we studied cell proliferation via metabolic activity by a metabolic assay and osteogenic differentiation via the activity of the tissue non-specific alkaline phosphatase enzyme (TNAP) at days, in culture after two and 11 days. 

## 2. Results and Discussion

### 2.1. Fibrils Characterization

**Figure 1:**
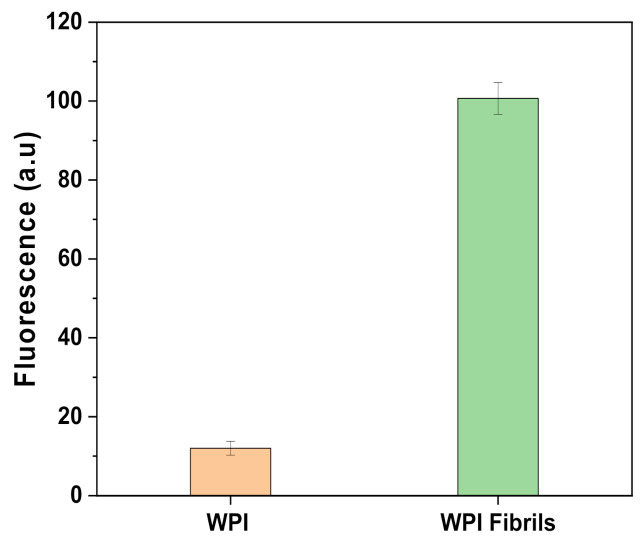
ThT test results of: 2.5% WPI at pH 2 (WPI); 2.5% WPI at pH 2 after 5 h incubation at 90 °C under stirring at 350 rpm (WPI Fibrils). λ_ex_ = 440 nm; λ_em_ = 486 nm. Error bars represent the standard deviation (SD).

The fluorescence emission of Thioflavin T (ThT) is an indication of the presence of fibrils since the molecule binds to β-sheets that are present in the protein [12]. The internal structure of the fibrils consists largely of crossed β-sheets arranged perpendicularly to the main axis of the fibrils. Figure 1 shows the results of the ThT assay made to assess if the fibrillation process has been successful. As clearly shown by the higher emission of the acid-hydrolysis treated sample (WPI fibrils) compared to the untreated sample (WPI), the reaction led to an increase of the fluorescence. A signal was also present in the untreated sample. This is certainly due to the presence of a small number of β-sheets prior to heat treatment, in accordance with Akkermans et al. [10]. Thereby, given the high increase in β-sheet numbers after heat treatment, the fibrillation reaction was successful.

**Figure 2:**
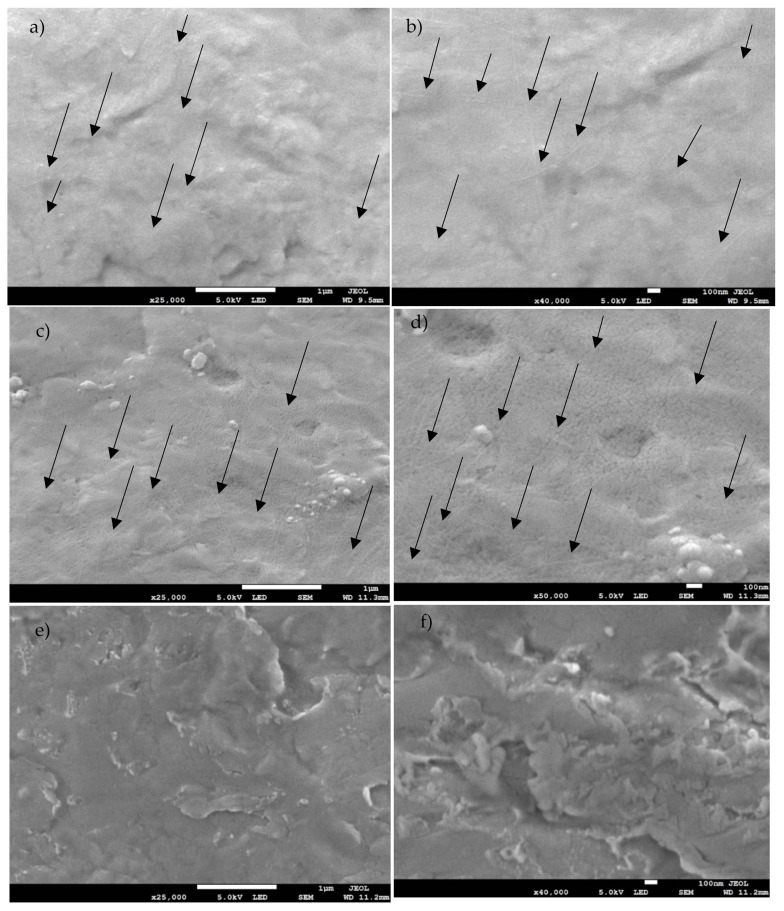
SEM images of Ti6Al4V WPI coated NS with 25,000× magnification (**a**) and 40,000× magnification (**b**); WPI coated S at 25,000× (**c**) and 50,000× (**d**); Ti6Al4V uncoated at 25,000× (**e**) and 40,000× (**f**). Black arrows indicate WPI fibrils. Scale bar: 1 µm for the 25,000× images and 0.1 µm for the more magnified.

The effective presence of the fibrils has been confirmed by the scanning electron microscopy (SEM) images shown in Figure 2. As indicated by the black arrows, on the coated not sterile (NS) (Figure 2a,b) and sterilized (S) (Figure 2c,d) samples there were long and thin fibrils well dispersed onto the analyzed area. The morphology appeared similar to the structure expected after fibrillation at pH 2 [13]. Moreover, the images further confirmed the ability of these type of fibrils to withstand washing and sterilization, as shown by previous work, in which glass substrates were used [14]. However, the presence of tinzaparin and heparin could not be detected by SEM. Therefore, the images are not shown in the article but can be found in the Supplementary Materials (S9–S10).

### 2.2. Coating Characterization

Table 1 shows the name of the samples object of this study and the corresponding treatment.

#### 2.2.1. Contact Angle

**Figure 3:**
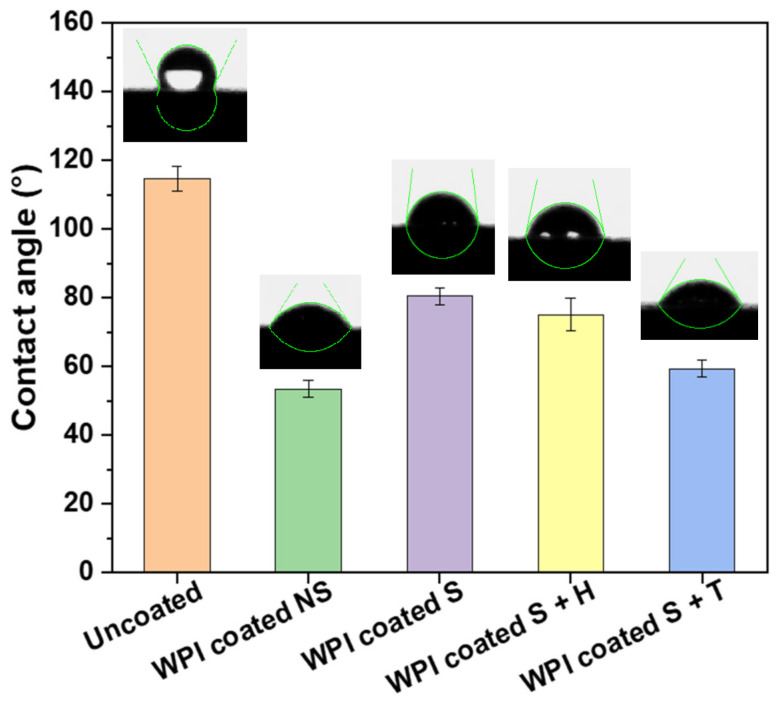
Contact angle measurements of Ti6Al4V discs: uncoated, coated with WPI fibrils at pH 2 not sterile (NS); coated with WPI fibrils at pH 2 and sterilized (S); coated and sterilized sample + heparin (S + H); coated and sterilized sample + tinzaparin (S + T). Error bars represent the standard deviation.

Figure 3 shows the contact angle of a 5 µL-MilliQ water drop on the surface of Ti6Al4V discs coated with WPI fibrils at pH 2 treated in different ways. Each sample was coated following the protocol described in Section 3.2. The uncoated sample (bare Ti6Al4V) showed a value of 114.73 ± 3.61°. On the other hand, the coated material showed a lower angle of contact in each condition: the sample “WPI Coated NS” had an angle of contact of 53.49 ± 2.35° while the same sample but sterilized (WPI Coated S) showed a slight increase in the value with 80.40 ± 2.31°. Finally, the samples with heparin and tinzaparin showed a value in between those for the “WPI Coated S” and “WPI Coated NS” of 75.11 ± 4.85° and 59.33 ± 2.45° respectively. The large reduction in the contact angle between the uncoated and coated pre-sterilization sample demonstrated a decrease in the hydrophobicity of the Ti6Al4V following coating with WPI fibrils.

According to statistical analysis (Table 2), each coated sample differed significantly from uncoated samples. However, among the coated samples, unsterilized and tinzaparin-enriched samples did not differ significantly in hydrophilicity. The same behavior can be observed between sterilized and heparin-enriched samples, which do not differ significantly from each other, but do differ from all other treatments. These results are in agreement with previous studies with titanium alloy [11] and WPI fibrils [14]. In addition, the surface became more hydrophobic following sterilization. One explanation could be that a small percentage of the coating was lost because of the heat treatment; however, further analyses are required. On the other hand, the addition of heparin made the sterilized sample more hydrophilic (as expected) from the polar nature of the molecule and the results of other studies on heparin-enriched coatings [15,16]. It is also important to note that the addition of tinzaparin increased hydrophilicity more than heparin, giving a first proof of the presence of the LMWH molecule. The increase of hydrophilicity leads to considerable advantages because a low hydrophobicity is required in orthopedic prostheses, especially if in contact with blood, as it reduces implant friction allowing better control in surgeries, limiting thrombosis and increasing general biocompatibility. The result is a reduction in the cost and time of the procedures [15].

#### 2.2.2. X-ray Photoelectron Spectroscopy

**Figure 4:**
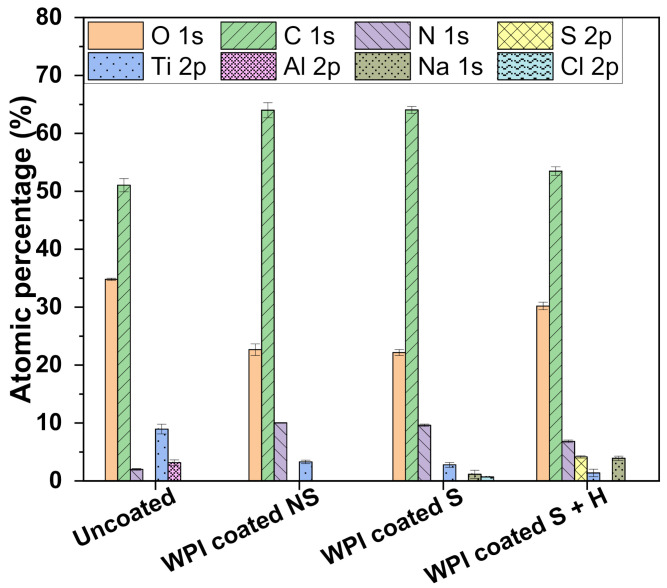
Atomic composition of uncoated and coated Ti6Al4V samples measured by XPS. Error bars represent the standard deviation.

X-ray photoelectron spectroscopy (XPS) results (Figure 4) showed that the superficial atomic composition of Ti6Al4V was: C (51%), O (34.8%), Ti (8.96%), Al (3.16%), N (2%). The WPI coated NS sample showed an increase in the amount of carbon and nitrogen, which are the main components of the coating. Consequently, the relative contents of oxygen and titanium decreased (22.6% and 3.3% respectively) accordingly. Weak signals of sodium (1.16%) and chlorine (0.71%) were recorded following sterilization (WPI coated S), which could be due to autoclave steam contamination since the instrument was loaded with tap water. Finally, it was possible to record the Sulphur signal (4.16%) in the sample further coated with heparin, indicating the actual presence of the molecule (survey and high-resolution spectra reported in Supplementary Figures S1–S8). However, with this technique, it was not possible to record the signal for the sample with tinzaparin. We assumed the presence of the LMWH molecule since in vitro investigations with hBMSC, e.g., the TNAP activity test on tinzaparin-coated samples, gave a higher result compared to the control and heparin-coated samples (see Section 2.4) and the CA measurement differed significantly among the heparin coated samples, as explained in the previous section. However, in future works it would be useful to develop a test to assess the presence of the molecule directly. In our preliminary work with WPI fibrils coatings, similar results were obtained with the same adsorption time on glass substrates [14], indicating that our protocol allows a good coating of the substrate with a thickness of at least 10 nm, which is considered to be the limit of detection of this technique.

### 2.3. Influence of Different Ti6Al4V Coating on Metabolic Activity of hBMSC

**Figure 5:**
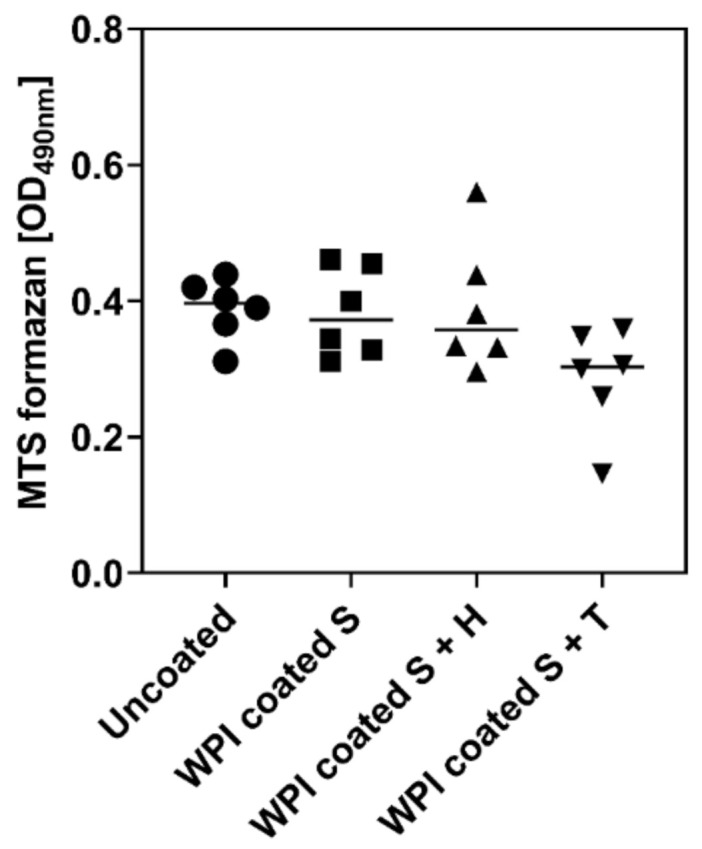
Metabolic activity of hBMSC on bare and coated Ti6Al4V. 6500 hBMSC/sample plated onto bare Ti6Al4V (uncoated) and Ti6Al4V coated with WPI + heparin, and WPI + tinzaparin and analysed after 48 h for metabolic activity. The results are shown as mean (bar) and individual values; *n* = 3. Statistically significant differences were not noted.

The MTS assay measures metabolic activity of cells as an indication for cell number and cell viability [17]. After two days in culture, the hBMSC number on coated Ti6Al4V seemed to be slightly lower than that on uncoated Ti6Al4V. In particular, the WPI-Tinzaparin coating tended to show the highest reduction of metabolic activity. However, the difference was not statistically significant (Figure 5). Hence, the coating had no impact on the number of cells that proliferate on the surface. In our previous study, similar results were found on glass-WPI coated samples showing that, even if the number of cells does not change after the coating, a better organization of the cytoskeleton occurs on the coated samples [14].

### 2.4. Influence of Different Ti6Al4V Coatings on TNAP Activity of hBMSC

**Figure 6:**
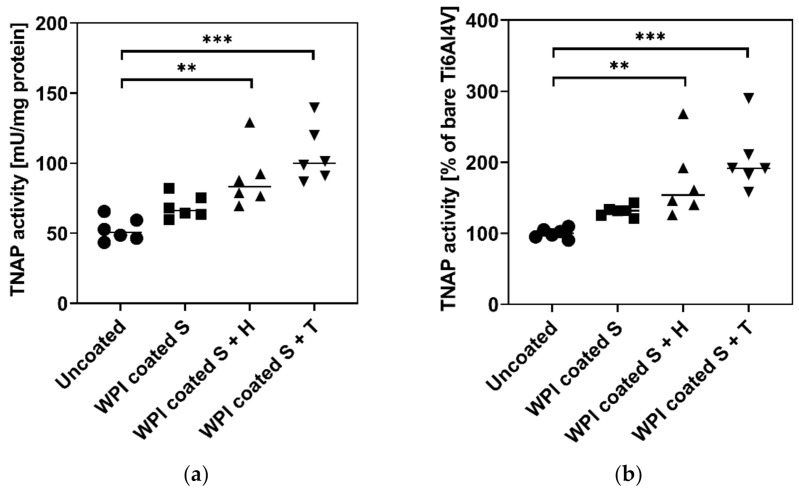
TNAP activity of hBMSC on bare and coated Ti6Al4V. 6500 hBMSC/sample plated in onto bare Ti6Al4V and Ti6Al4V coated WPI, WPI + heparin, and WPI + tinzaparin. From day four after plating, the cells were cultured with osteogenic supplements and analysed at day 11 for TNAP enzyme activity. The results are shown as mean (bar) and individual values; *n* = 3. Statistical significant differences versus bare Ti6Al4V are indicated with ** (*p* < 0.01) and *** (*p* < 0.001). (**a**) mU/mg protein; (**b**) % of bare Ti6Al4V.

For evaluation of the osteoinductive potential of different Ti6Al4V coatings, the activity of the TNAP enzyme in hBMSC cultured for 11 days was analyzed. Besides the deposition of calcium phosphate as a final differentiation marker for osteoblasts, TNAP activity is frequently used as an early osteogenic differentiation marker that indicates the potential of hBMSC to form hydroxyapatite [18,19]. Thus, TNAP activity is a prerequisite for bone mineralization since the ectoenzyme releases phosphate ions and therefore increases their concentration locally so that, finally, hydroxyapatite can be formed. The higher the activity of alkaline phosphatase (ALP), the more bone mineral is deposited and the better an implant can be integrated [20,21]. As highlighted in Figure 6, the coating of Ti6Al4V with WPI slightly increased TNAP activity. The addition of heparin and tinzaparin to the WPI significantly increased the activity of TNAP at day 11. The higher effect of tinzaparin could be related to its short chain length compared to heparin. These findings were consistent with our previous work which showed the significant effect of WPI coating on the activity of TNAP [14]. However, here we proved that this behavior can be further enhanced by adding LMWH to the system. Simann et al. [22] and Mathews et al. [23] both showed a higher activity of ALP in hBMSC cultured in the presence of heparin, supporting our results. Additionally, in our previous study, the positive effect of heparin on the early-stage osteoblast-differentiation occurred only when the molecule was bound to a protein substrate [24]. The results showed in this work gave a clear indication of a pro-osteogenic effect of LMWH-modified WPI implant coatings. These results are a promising starting point for future investigations involving more osteoblast features and osteogenic parameters as mineral deposition, formation of osteocalcin, osteoprotegerin, osteopontin, bone sialoprotein, etc. in order to elucidate the molecular mechanisms of such coatings and to characterize the effects of heparin and tinzaparin as coating components in more detail.

## 3. Materials and Methods

### 3.1. Fibrils Production and Characterization

WPI fibrils were prepared according to the protocol described by Keppler et al. [25]): WPI (BiPro, Davisco Foods International Inc., Eden Prairie, MN, USA) was dissolved in Milli-Q water to a final concentration of 2.5 wt% and the pH was set to 2.0 with 2M HCl. 40 mL of protein solution were heated at 90 °C for five hours under stirring at 350 rpm to allow the fibrillation reaction to take place. At the end of the specified time, the solution was immediately cooled on ice to stop the reaction. The fibrils were stored in a refrigerator and proved to be stable for approximately four months.

Qualitative determination of the fibrils was carried out using the ThT colorimetric assay described by Loveday et al. [26]: 12 μL of the protein solution was added to 1 mL of ThT (Sigma-Aldrich, Schnelldorf, Germany) solution and fluorescence was measured with a plate reader (Infinite M200 PRO; Tecan, Reading, UK) after 1 min of incubation directly in a 96-well plate. Excitation and emission wavelengths were 420 nm and 486 nm, respectively. The analysis has been conducted against a blank sample with water and ThT.

### 3.2. Coating Protocol and Characterization

The Ti6Al4V discs (2 cm of diameter and 1 mm thick) additively manufactured in an A2 ARCAM EBM machine (ARCAM EBM, Mölnlycke, Sweden) were coated with fibrils by adsorption from the suspension. The substrates were left in contact with 1 mL of the fibril solution (2.5 wt%) for one hour, then rinsed three times with Milli-Q water to remove excess coating and left to air dry. For samples with heparin (or tinzaparin), the protocol was repeated on the fibril-coated samples using a 10 wt% heparin (or tinzaparin) solution.

The sterilization (121 °C, 15 min, 1 atm) was performed after the coating procedure with a Bench top Autoclave. Addition of heparin or tinzaparin by adsorption from solutions was performed in sterile conditions under a laminar flow hood using syringe (NORM-JECT 12 mL, Henke-Sass Wolf GmbH, Tuttlingen, Germany) and filters (28 mm Diameter Syringe Filters, 0.2 µm Pore SFCA-PF Membrane, Corning International, New York, NY, USA).

X-ray photoelectron spectroscopy (XPS) was performed to analyze the surface chemical composition on the samples using an Axis Supra spectrometer (Kratos Analytical Ltd., Manchester, UK) with a monochromatic Al Kα source (1.487 keV). Samples were mounted using carbon tape on a sample holder. An internal flood gun was used for neutralizing charging effects. Wide scans were recorded at a pass energy of 160 eV and a step size of 1 eV. Samples were measured at an emission angle of 0° (relative to the surface normal), power of 225 W (15 kV × 15 mA) and an analysis area of 700 × 300 µm. Three different locations on each coating’s type were analyzed. Spectra were analyzed with CasaXPS software (version 2.3.22, Casa Software Ltd., Devon, UK). All binding energies were referenced to the C-C component of the C1s spectrum at 284.8 eV to compensate for the surface charging effects. The curve fitting procedure of the components was performed using Gaussian-Lorentzian function and a linear background.

Contact angle measurements were achieved with a home-built system consisting of a light, a stand and a camera connected to a computer. The images were analysed with ImageJ software with the drop analysis plugin [27].

The SEM characterization was performed as follows: samples were mounted on standard aluminum pin stubs using double sided conductive carbon adhesive dots. They were subsequently sputter coated with approximately 5 nm of gold (at 20 mA for 60 s, 1x 10-2 mBar, under argon) using a system by Quorum Technologies Ltd., Lewes, UK, Q150RES. Finally, samples were imaged using a Jeol JSM-7800F Field Emission Scanning Electron Microscope (FEG-SEM) using the lower secondary electron detector.

### 3.3. Isolation and Cultivation of Human Bone Marrow Stromal Cells (hBMSC)

hBMSC were isolated from bone marrow aspirates obtained from donors at the Bone Marrow Transplantation Center of the University Hospital Dresden. The cells were characterized as described in the work by [28]. The donors (males, average age 25 ± 3 years.) were duly informed about the procedures and gave their full consent. The study was approved by the local ethics commission (ethic vote No. EK466112016).

For the in vitro experiments, 5000 hBMSC were seeded in an 80 µL-droplet of cell culture medium (Dulbecco’s minimal essential medium (DMEM; Merck-Millipore, Darmstadt, Germany), supplemented with 10% heat-inactivated fetal calf serum and antibiotics (Sigma-Aldrich,) onto the surface of each sample (Ø 10 mm, 1 mm height, 0.78 cm^2^). Two hours after plating, DMEM was added to cover the samples with medium. At day four after plating, cell culture medium was replaced by osteogenic differentiation medium (DMEM with 10% heat-inactivated fetal calf serum and antibiotics supplemented with 10 mM β-glycerol phosphate, 300 µM ascorbate, and 10 nM dexamethasone (Sigma-Aldrich) as described previously [29]. The medium was changed twice per week.

### 3.4. Determination of Metabolic Activity

The metabolic activity of hBMSC cells was determined by the MTS assay (Cell Titer96 AQ_ueous_ One Solution Proliferation Assay; Promega, Germany) at day two after plating. The cell culture medium was replaced by fresh medium containing 10% of MTS dye solution. After 2 h of incubation at 37 °C in a humidified CO_2_ incubator, 80 µL of medium was transferred into a 96-well plate and the absorbance of the formed formazan dye was measured photometrically at 490 nm.

### 3.5. Determination of Tissue Non-Specific Alkaline Phosphatase (TNAP) Enzyme Activity

At day 11 after seeding, hBMSC were analysed for TNAP enzyme activity. TNAP enzyme activity was determined from cell lysates (TNAP lysis buffer: 1.5 M Tris-HCl, pH 10 containing 1 mM ZnCl_2_, 1 mM MgCl_2_ and 1% Triton X-100; Sigma-Aldrich, Germany) with p-nitrophenylphosphate (Sigma-Aldrich, Germany) as a substrate, as previously described [30]. TNAP activity was calculated from a linear calibration curve (r > 0.99) prepared with p-nitrophenolate. Protein concentration of the lysate was determined with RotiQuant protein assay (Roth GmbH, Karlsruhe, Germany) and was calculated from a linear calibration curve (r > 0.99) obtained with bovine serum albumin (Serva, Heidelberg, Germany). Specific TNAP activity is given in mU/mg protein.

### 3.6. Statistical Analysis

Cell experiments were performed with cells from three different donors (*n* = 3) each in duplicate. The results were presented as mean ± standard error of the mean (SEOM). Statistical significance was analyzed with GraphPad Prism 8.4 software (Statcon, Witzenhausen, Germany) by ANOVA analysis with Bonferroni’s post-test. The contact angle was measured on four water drops for each sample. One-way ANOVA with Tukey’s post-test was carried out with the software IBM SPSS Statistics Version 27.

## 4. Conclusions

Coating Ti6Al4V with WPI fibrils obtained at pH 2 enriched with heparin and tinzaparin proved to be a successful strategy to create a viable substrate for hBMSC. This substrate for the cells promotes osteogenic differentiation by improving the quality of the differentiated cells, as evidenced by the increase in the TNAP activity. In particular, this work showed that enriching the coating with heparin and tinzaparin improves the aforementioned effect considerably. Specifically, it seems that tinzaparin has the highest impact on the TNAP activity. In any case, further investigations of the molecular mechanism are needed to further elucidate this behavior. As far as the coating protocol is concerned, one hour of adsorption time was sufficient to successfully coat Ti6Al4V substrates. However, the presence of some uncoated areas was detected. Additionally, XPS can be used as a method to evaluate the presence of heparin and proteins on the surface of the material. Nonetheless, it seems that this is not a suitable method for the detection of tinzaparin, and it advisable to use a different approach in future work. The coating increased the hydrophilicity of the material with a higher extent when enriched with tinzaparin compared to heparin. This behavior, together with the statistically significant difference seen between the heparin and tinzaparin coated samples in the TNAP activity test, provides further evidence of the presence of the LMWH molecule.

## Figures and Tables

**Table 1:** Samples name and treatment.

Sample Name	Treatment
Uncoated	Bare Ti6Al4V
WPI Coated NS (Not Sterile)	Ti6Al4V coated with WPI fibrils
WPI Coated S (Sterile)	Ti6Al4V coated with WPI fibrils and autoclaved
WPI Coated S + H (Sterile + Heparin)	Ti6Al4V coated with WPI fibrils, autoclaved and subsequently coated with Heparin
WPI Coated S + T (Sterile + Tinzaparin)	Ti6Al4V coated with WPI fibrils, autoclaved and subsequently coated with Tinzaparin (LMWH)

**Table 2:** Water-Contact angle test results on Ti6Al4V uncoated and coated in different conditions. Results are expressed as mean ± standard deviation (SD). Means without a common superscript differ (*p* < 0.05).

Sample	Mean ± SD (°)
Uncoated	114.73 ± 3.61 ^a^
WPI Coated NS	53.49 ± 2.35 ^be^
WPI Coated S	80.40 ± 2.31 ^cd^
WPI Coated S + H	75.11 ± 4.85 ^cd^
WPI Coated S + T	59.33 ± 2.45 ^be^

## Data Availability

Not applicable.

## Supplementary Materials

The following are available online at https://www.mdpi.com/article/10.3390/ijms23010139/s1.

## Abbreviations

AM	Additive Manufacturing
ALP	Alkaline phosphatase
CA	Contact angle
DMEM	Dulbecco’s minimal essential medium
FEG-SEM	Field Emission Scanning Electron Microscope
GAGs	Glycosaminoglycans
hBMSC	human bone marrow stromal cells
LMWH	Low molecular weight heparin
NS	Not sterile
S	Sterile
SD	Standard deviation
SEOM	Standard error of the mean
SEM	Scanning Electron Microscopy
ThT	Thioflavin T
TNAP	Tissue non-specific alkaline phosphatase
WPI	Whey protein isolate
β-lg	β-lactoglobulin
