# Supplementary material for: Heparin Enriched-WPI Coating on Ti6Al4V Increases Hydrophilicity and Improves Proliferation and Differentiation of Human Bone Marrow Stromal Cells
Source: Int J Mol Sci. 2021 Dec 23;23(1):139. doi: 10.3390/ijms23010139 (PMC8745389; doi:10.3390/ijms23010139)
Supplement: Supplementary file 1 [file ijms-23-00139-s001.zip › ijms-1477752-supplementary.pdf]

Supplementary figures of "Heparin enriched-WPI coating on Ti6Al4V increases hydrophilicity and improves proliferation and differentiation of human bone marrow stromal cells"

Davide Facchetti [1,3*], Ute Hempel [2], Laurine Martocq [1], Alan M. Smith [4], Andrey Kopytug [5], Roman A. Surmenev [6], Maria A. Surmeneva [6], and Timothy E.L. Douglas [1,7*]

[1] Engineering Department, Lancaster University, Lancaster, United Kingdom;
[2] Institute of Physiological Chemistry, Technische Universität Dresden, Germany;
[3] Department of Chemistry, Molecular Sciences Research Hub, Imperial College London, United Kingdom;
[4] Department of Pharmacy, School of Applied Sciences, University of Huddersfield, United Kingdom;
[5] Department of Quality Technology, Mechanical Engineering & Mathematics, Mid Sweden University, 831 25 Östersund, Sweden;
[6] Physical Materials Science and Composite Materials Centre, Research School of Chemistry & Applied Biomedical Sciences, National Research Tomsk Polytechnic University, 634050 Tomsk, Russia;
[7] Materials Science Institute (MSI), Lancaster University, United Kingdom

Correspondence: * D.F: d.facchetti21@imperial.ac.uk;T.E.L.D: t.douglas@lancaster.ac.uk

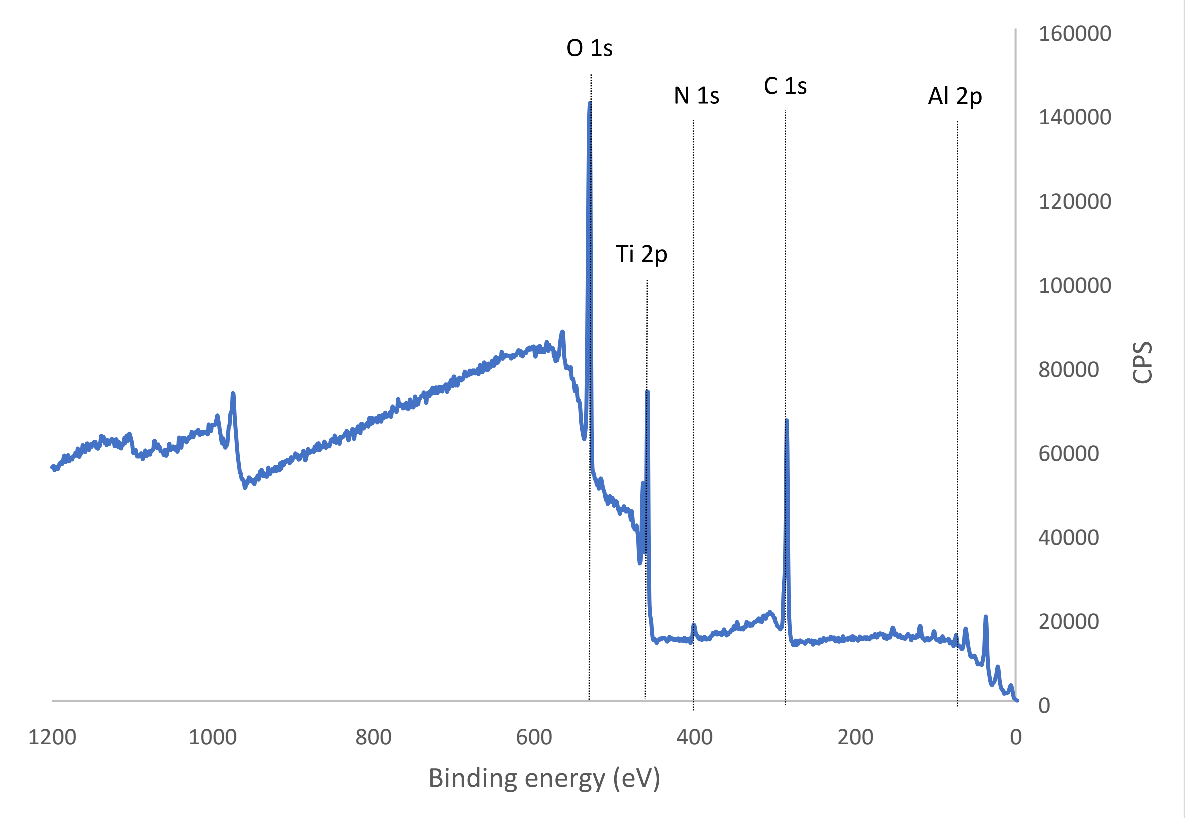

Supplementary Figure S1. XPS Survey spectrum of bare Ti6Al4V (Uncoated sample)

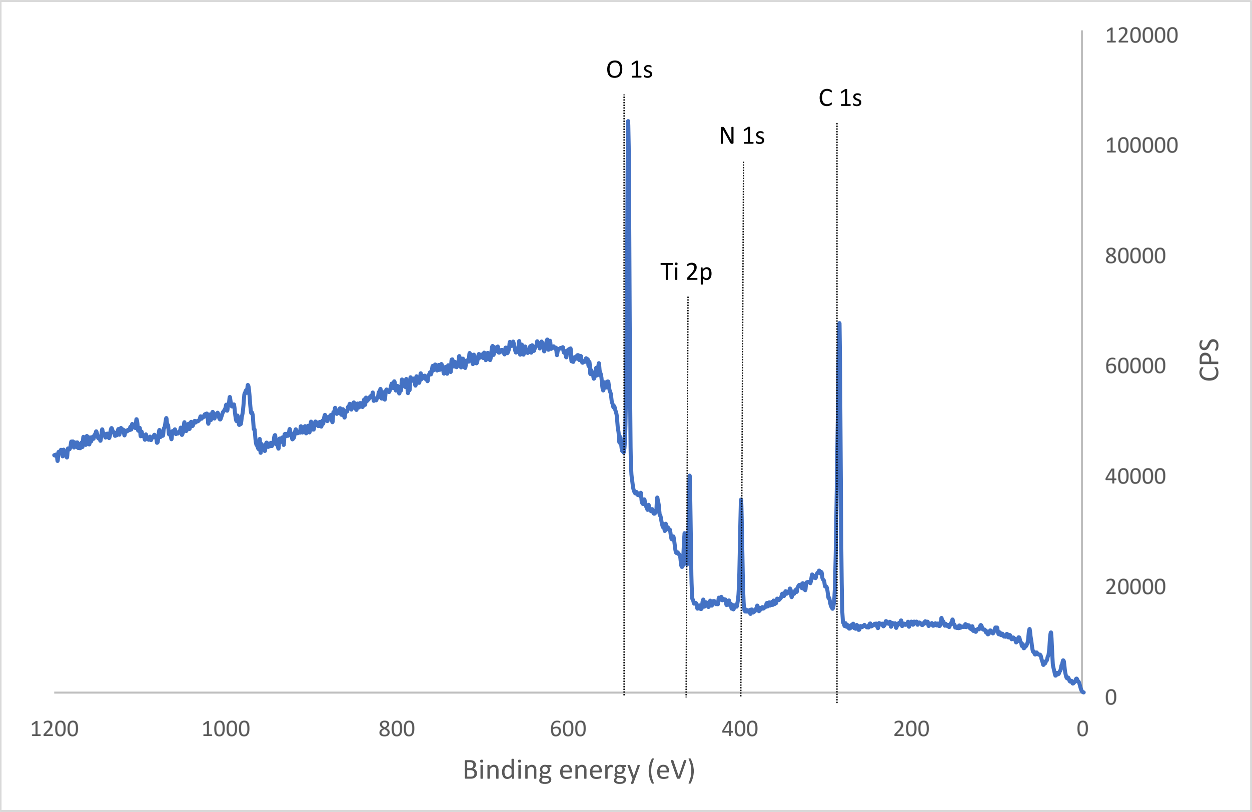

Supplementary Figure S2. XPS Survey spectrum of Ti6Al4V WPI coated NS

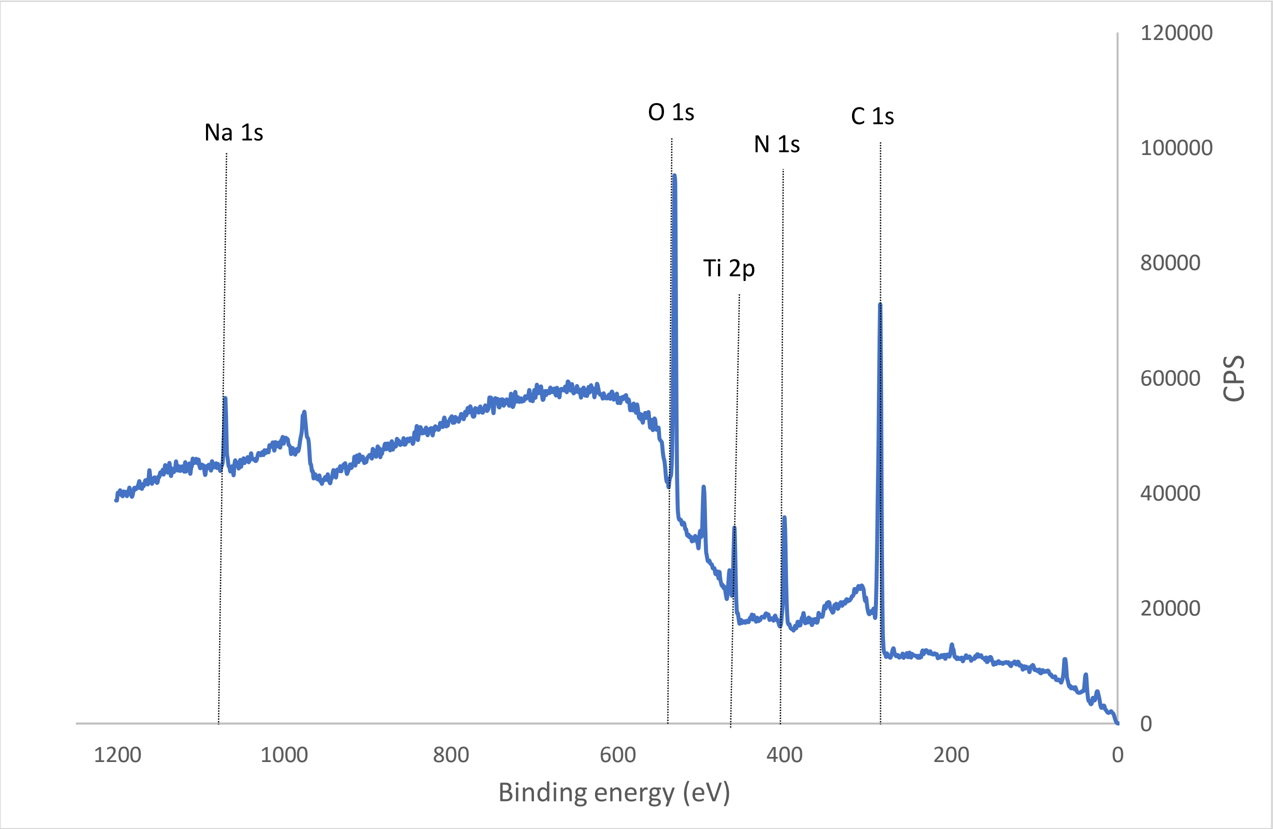

Supplementary Figure S3. XPS Survey spectrum of Ti6Al4V WPI Coated S

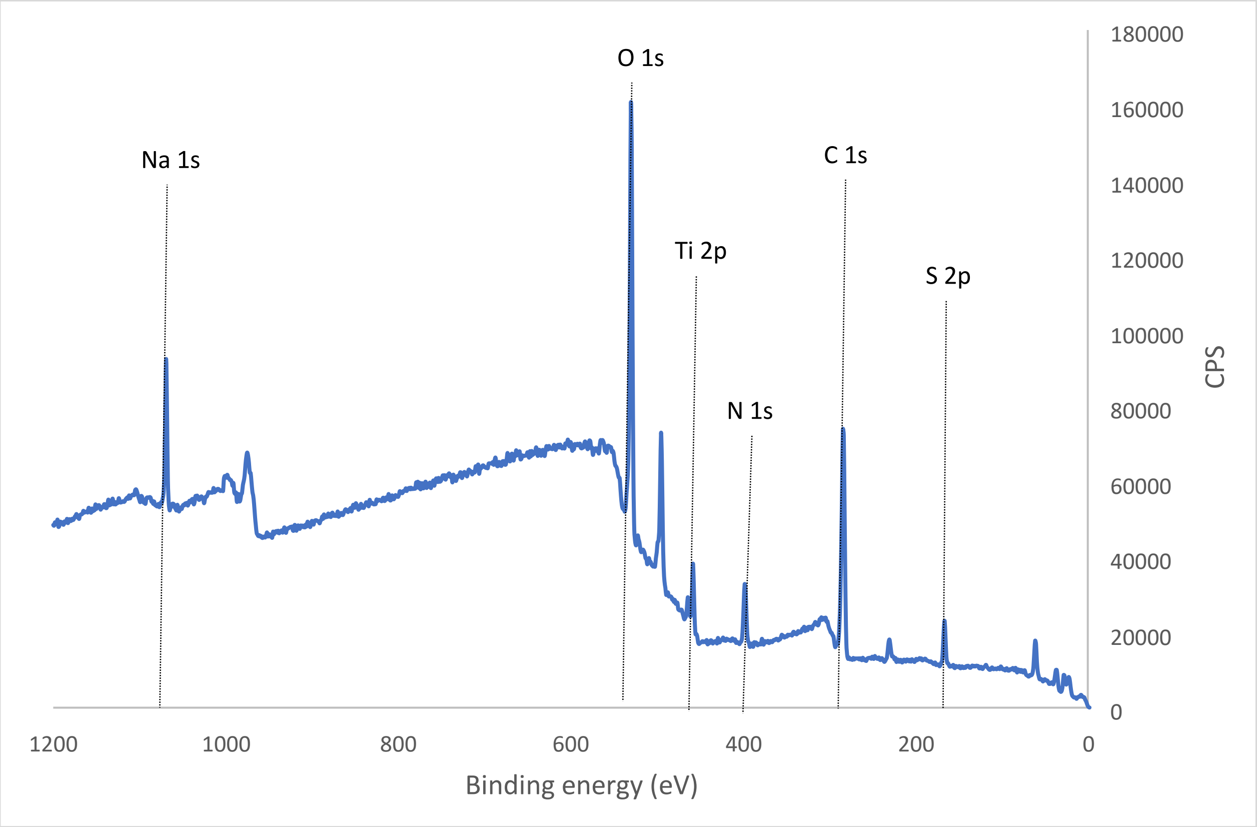

Supplementary Figure S4. XPS Survey spectrum of Ti6Al4V WPI Coated S + H

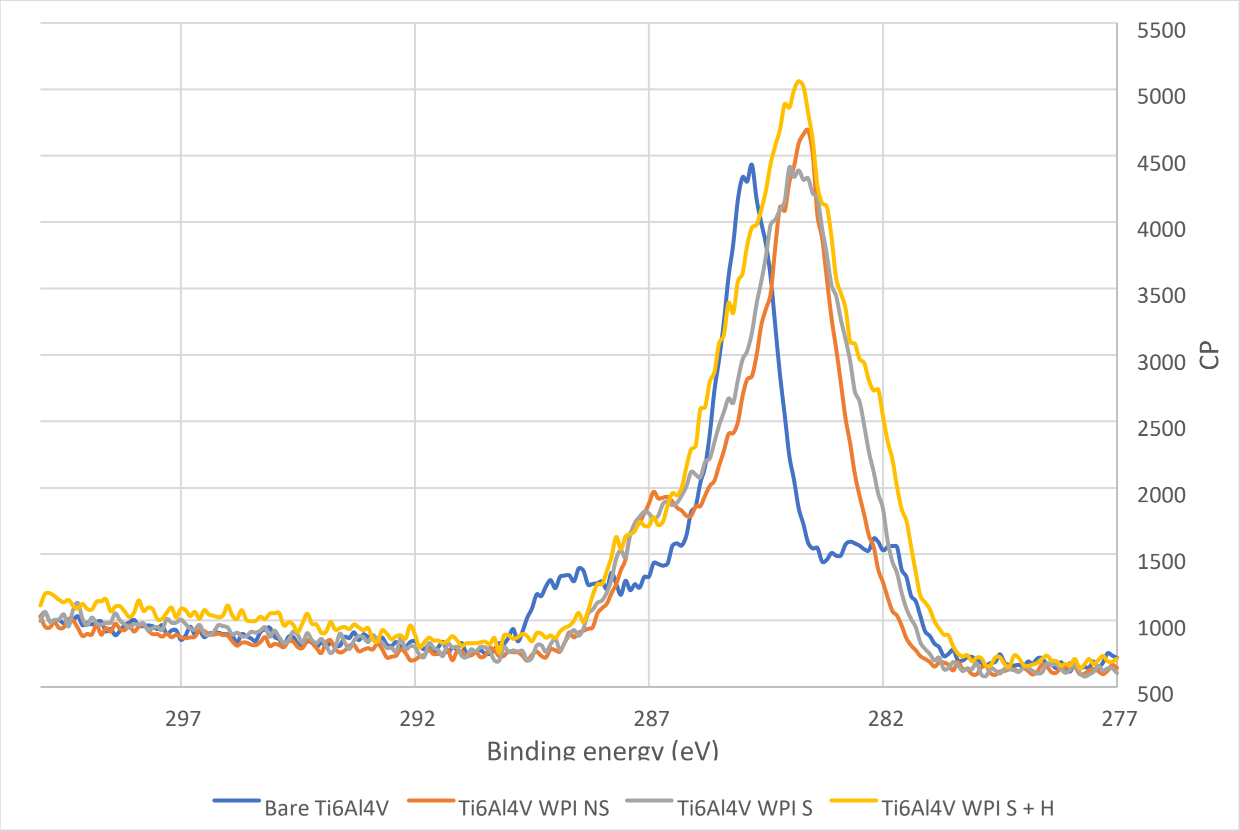

Supplementary Figure S5. XPS high resolution spectrum of Carbon

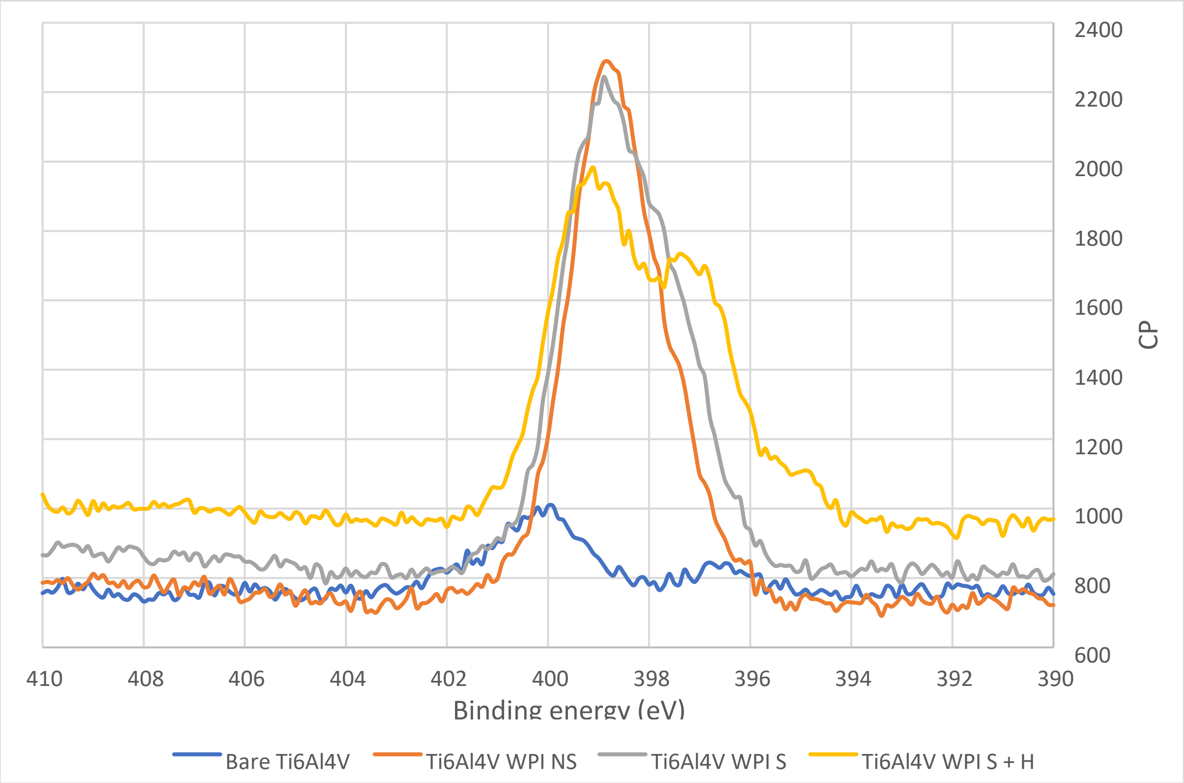

Supplementary Figure S6. XPS high resolution spectrum of Nitrogen

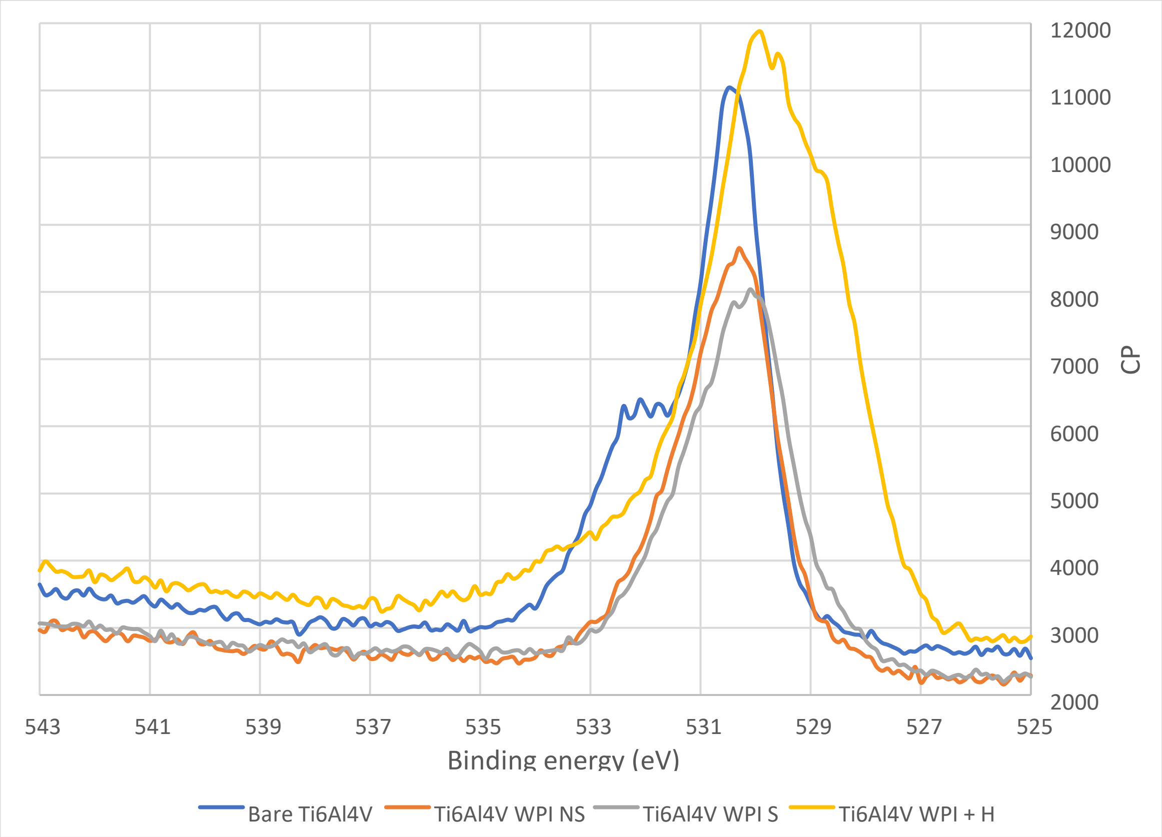

Supplementary Figure S7. XPS high resolution spectrum of Oxygen

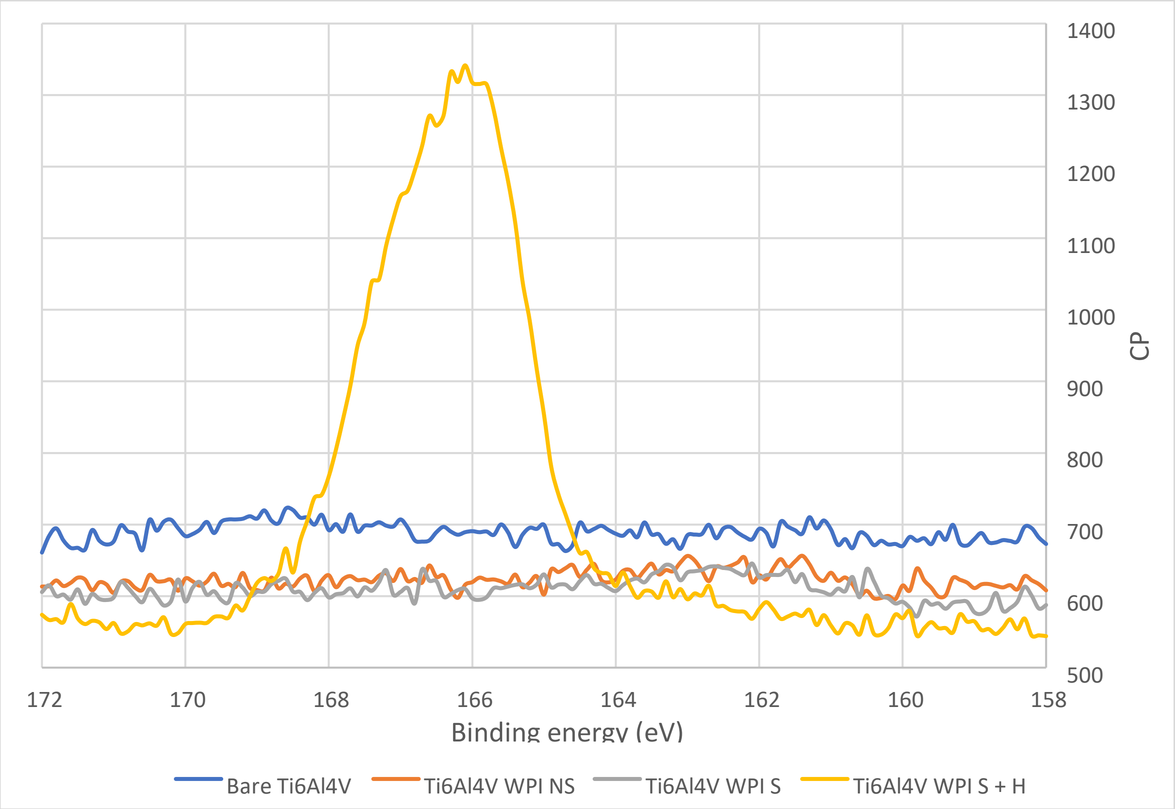

Supplementary Figure S8. XPS high resolution spectrum of Sulfur

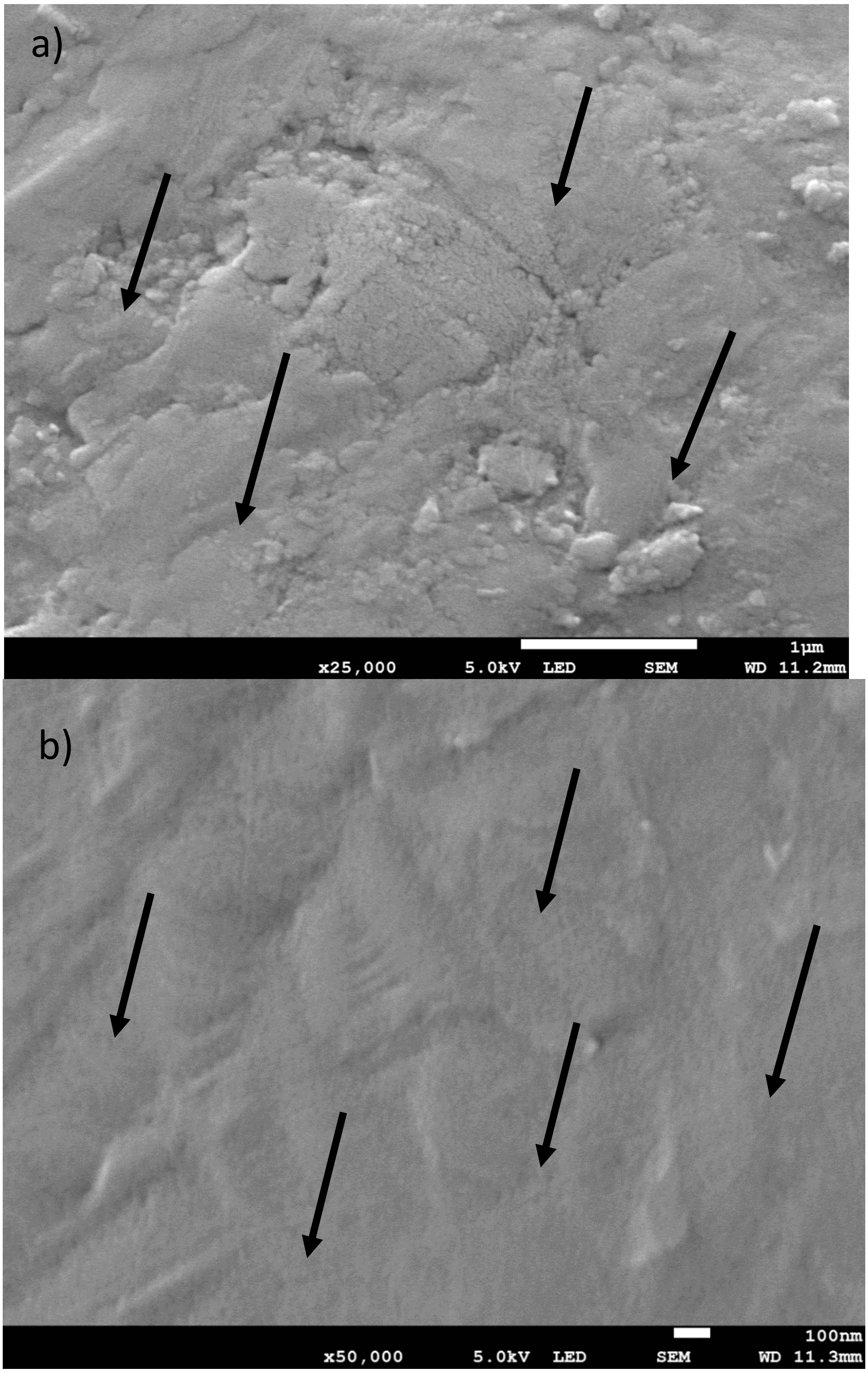

Supplementary Figure S9. SEM images of Ti6Al4V Coated S + H. Black arrows indicate WPI fibrils. No clear evidence of heparin. At both 25000 (a) and 50000 (b) magnification respectively.

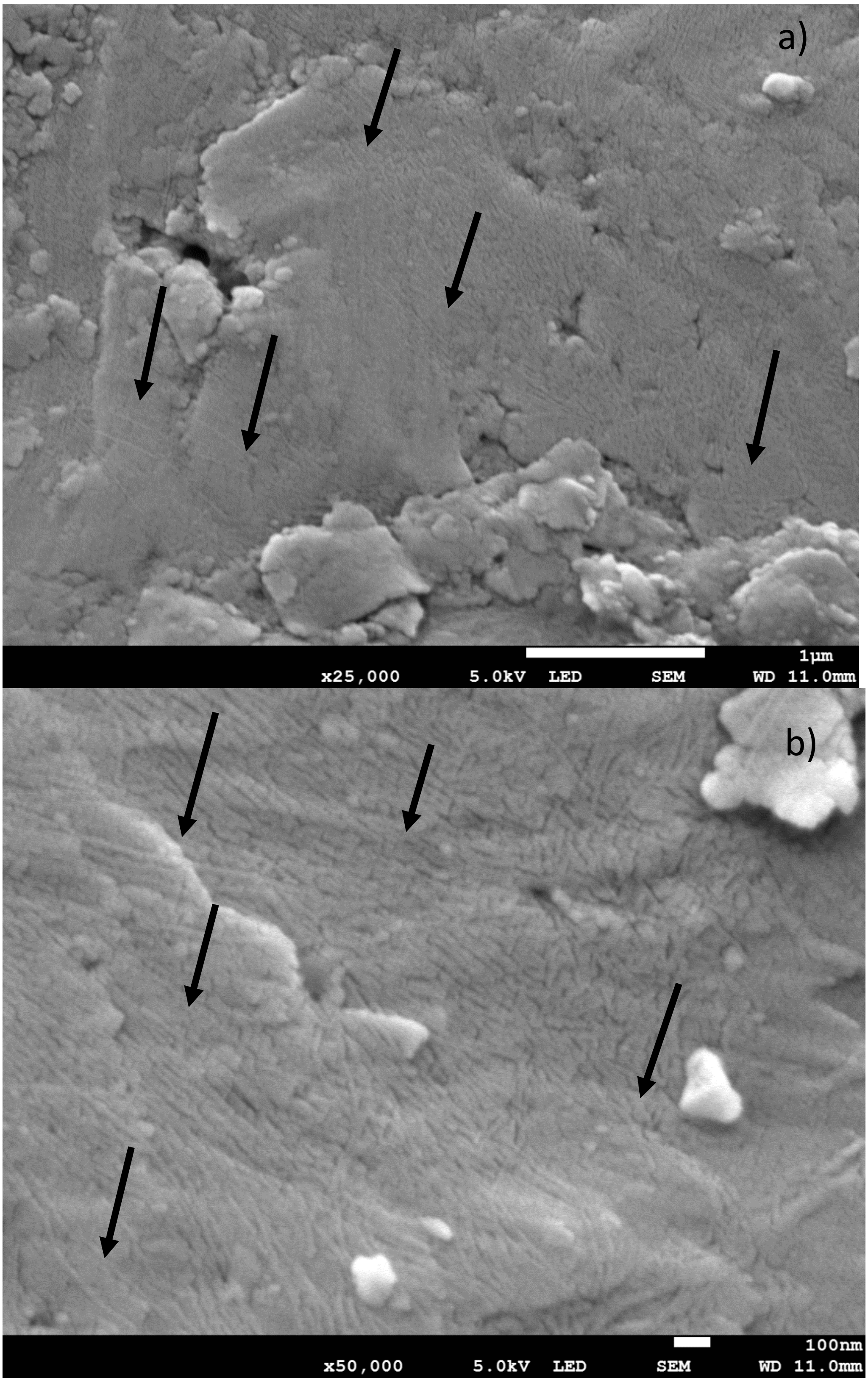

Supplementary Figure S10. SEM images of Ti6Al4V Coated S + T. Black arrows indicate WPI fibrils. No clear evidence of tinzaparin. At both 25000 (a) and 50000 (b) magnification respectively.

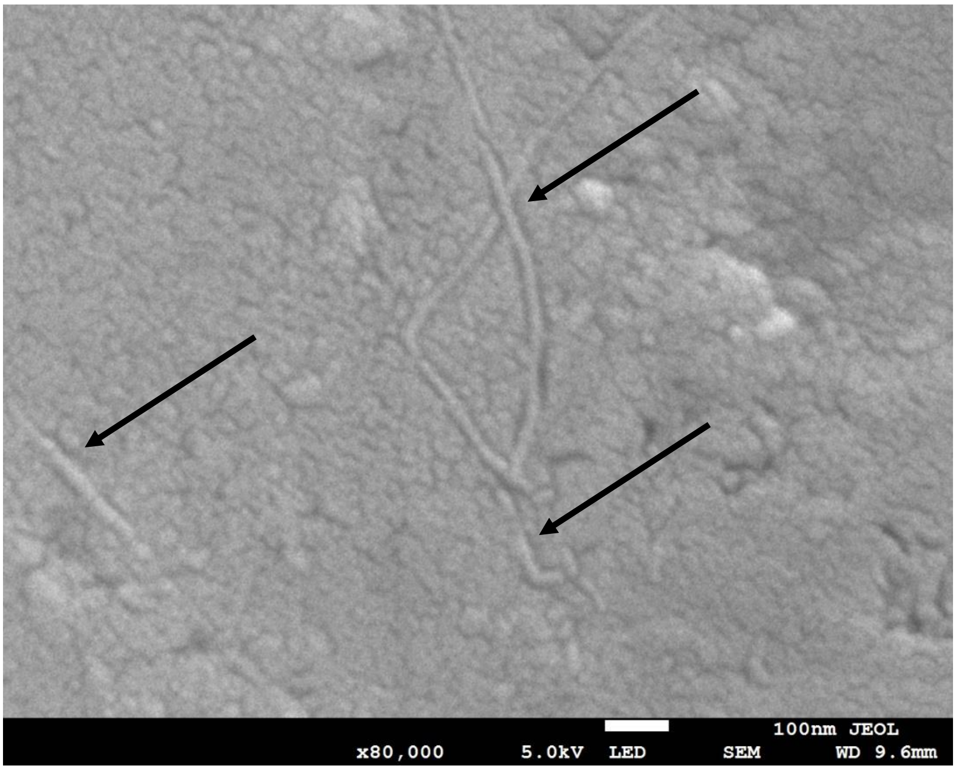

Supplementary Figure S11. SEM images of Ti6Al4V coated NS at 80000x magnification. Black arrows indicate WPI fibrils
